# Plasma Membrane Calcium ATPase-Neuroplastin Complexes Are Selectively Stabilized in GM1-Containing Lipid Rafts

**DOI:** 10.3390/ijms222413590

**Published:** 2021-12-18

**Authors:** Katarina Ilic, Xiao Lin, Ayse Malci, Mario Stojanović, Borna Puljko, Marko Rožman, Željka Vukelić, Marija Heffer, Dirk Montag, Ronald L. Schnaar, Svjetlana Kalanj-Bognar, Rodrigo Herrera-Molina, Kristina Mlinac-Jerkovic

**Affiliations:** 1 Croatian Institute for Brain Research, School of Medicine, University of Zagreb, 10000 Zagreb, Croatia; ilic.katarina@mef.hr (K.I.); mario.stojanovic@mef.hr (M.S.); borna.puljko@mef.hr (B.P.); svjetlana.kalanj.bognar@mef.hr (S.K.-B.); 2 BRAIN Centre, Department of Neuroimaging, Institute of Psychiatry, Psychology and Neuroscience (IOPPN), King’s College London, London SE5 9NU, UK; 3 Neurogenetics Laboratory, Leibniz Institute for Neurobiology, 39118 Magdeburg, Germany; xlin@lin-magdeburg.de (X.L.); Dirk.Montag@lin-magdeburg.de (D.M.); 4 Synaptic Signalling Laboratory, Combinatorial NeuroImaging, Leibniz Institute for Neurobiology, 39118 Magdeburg, Germany; Ayse.Malci@lin-magdeburg.de (A.M.); Rodrigo.Herrera-Molina@lin-magdeburg.de (R.H.-M.); 5 Department of Chemistry and Biochemistry, School of Medicine, University of Zagreb, 10000 Zagreb, Croatia; zeljka.vukelic@mef.hr; 6 Department of Physical Chemistry, Ruđer Bošković Institute, 10000 Zagreb, Croatia; marko.rozman@irb.hr; 7 Department of Medical Biology and Genetics, Faculty of Medicine, University of Osijek, 31000 Osijek, Croatia; marija.heffer@mefos.hr; 8 Departments of Pharmacology and Neuroscience, Johns Hopkins University School of Medicine, Baltimore, MD 21205, USA; schnaar@jhu.edu; 9 Centro Integrativo de Biología y Química Aplicada, Universidad Bernardo O’Higgins, Santiago 8307993, Chile; 10 Center for Behavioral Brain Sciences, 39120 Magdeburg, Germany

**Keywords:** neuronal calcium homeostasis, gangliosides, glycosphingolipids, GM2/GD2 synthase, *B4galnt1*, membrane microdomains

## Abstract

The recent identification of plasma membrane (Ca^2+^)-ATPase (PMCA)-Neuroplastin (Np) complexes has renewed attention on cell regulation of cytosolic calcium extrusion, which is of particular relevance in neurons. Here, we tested the hypothesis that PMCA-Neuroplastin complexes exist in specific ganglioside-containing rafts, which could affect calcium homeostasis. We analyzed the abundance of all four PMCA paralogs (PMCA1-4) and Neuroplastin isoforms (Np65 and Np55) in lipid rafts and bulk membrane fractions from GM2/GD2 synthase-deficient mouse brains. In these fractions, we found altered distribution of Np65/Np55 and selected PMCA isoforms, namely PMCA1 and 2. Cell surface staining and confocal microscopy identified GM1 as the main complex ganglioside co-localizing with Neuroplastin in cultured hippocampal neurons. Furthermore, blocking GM1 with a specific antibody resulted in delayed calcium restoration of electrically evoked calcium transients in the soma of hippocampal neurons. The content and composition of all ganglioside species were unchanged in Neuroplastin-deficient mouse brains. Therefore, we conclude that altered composition or disorganization of ganglioside-containing rafts results in changed regulation of calcium signals in neurons. We propose that GM1 could be a key sphingolipid for ensuring proper location of the PMCA-Neuroplastin complexes into rafts in order to participate in the regulation of neuronal calcium homeostasis.

## 1. Introduction

The plasma membrane (PM) separates the intra- and extra-cellular environments. Positioning and function of membrane proteins in the PM is strongly influenced by dynamically changing lipid composition and interactions. The PM is not a simple nor a homogeneous milieu, but it is a highly dynamic structure resembling a patchwork of lipid raft microdomains with distinct composition and chemical properties. These nanometer-scale submembrane compartments are enriched with (glyco)sphingolipids, cholesterol, and a specific subset of transmembrane proteins [1,2]. Hence, lipid-protein interdependence and functional interplay is markedly highlighted in these domains. One family of transmembrane proteins for which fine-tuning of localization and activity is associated with lipid rafts is the Plasma Membrane (Ca^2+^)-ATPase (PMCA) family. PMCA removes Ca^2+^ from the cell cytosol at the expense of ATP, being one of the main regulators of the intracellular Ca^2+^ concentration [3]. Amongst many functions fundamental to excitable cells such as neurons, controlled Ca^2+^ exchange between the extracellular space (mM Ca^2+^ concentration) and the cytoplasm (μM Ca^2+^ concentration) is a prerequisite for normal neuronal function. Calcium signaling regulates many cell processes including neurotransmitter release, synaptic plasticity, gene expression and cell survival to name a few. Therefore, changes of Ca^2+^ homeostasis can result in profound functional alterations [4,5].

Recent reports demonstrated that approximately 95% of PMCA is associated with Neuroplastin (Np) [6,7,8]. The two Neuroplastin isoforms (Np65 and Np55) are heavily glycosylated transmembrane proteins and members of the immunoglobulin-superfamily of cell adhesion molecules. Neuroplastin has been involved in synaptic plasticity, is essential for cognition, long-term potentiation, and associative memory formation, in addition to being implicated in Alzheimer’s disease [9,10,11,12,13,14,15,16,17,18,19,20]; for a recent review see Ilic et al. 2021 and Lin et al. 2021 [19,20]. We reported that Neuroplastin expression is altered in the brains of mice lacking complex gangliosides [21], using a genetically engineered mouse model that lacks the GM2/GD2 synthase enzyme. The GM2/GD2 synthase, coded by the *B4galnt1* gene, is responsible for synthesizing a- and b-series gangliosides, including GM1, GD1a, GD1b and GT1b which constitute 95% of all gangliosides in the mammalian brain [22,23]. The association of PMCA with rafts [24,25,26,27] and our research on the influence of gangliosides on expression and localization of Neuroplastin [21] suggests that PMCA-Np complexes may be affected by gangliosides.

Here, we investigated the hypothesis that the lipid environment in rafts determined by gangliosides is important for the presence and function of PMCA-Neuroplastin complexes in specific nanodomains. We analyzed the submembrane localization of PMCA and Neuroplastin in brain tissue of the GM2/GD2 synthase-deficient mouse model, examined the co-localization of Neuroplastin with main brain gangliosides in primary neuronal cultures, evaluated calcium transients in primary neuronal cultures treated with anti-ganglioside antibodies, and investigated the brain ganglioside composition in Neuroplastin-deficient mice. Using this comprehensive approach, we discovered that disorganization of GM1 ganglioside-containing rafts causes perturbation in PMCA-Np functionality and results in altered regulation of calcium signals in neurons.

## 2. Results

### 2.1. Content of Neuroplastin and PMCAs Is Altered in Lipid Rafts from GM2/GD2 Synthase-Deficient Mice

In order to evaluate the effect of ganglioside composition on the exact submembrane localization of Neuroplastins and PMCAs, i.e., their abundance in lipid rafts (LR) and the bulk membrane (non-lipid raft; non-LR), we performed lipid raft analysis isolation and Western blotting analysis of Nps and PMCAs expression in individual membrane fractions.

**Figure 1:**
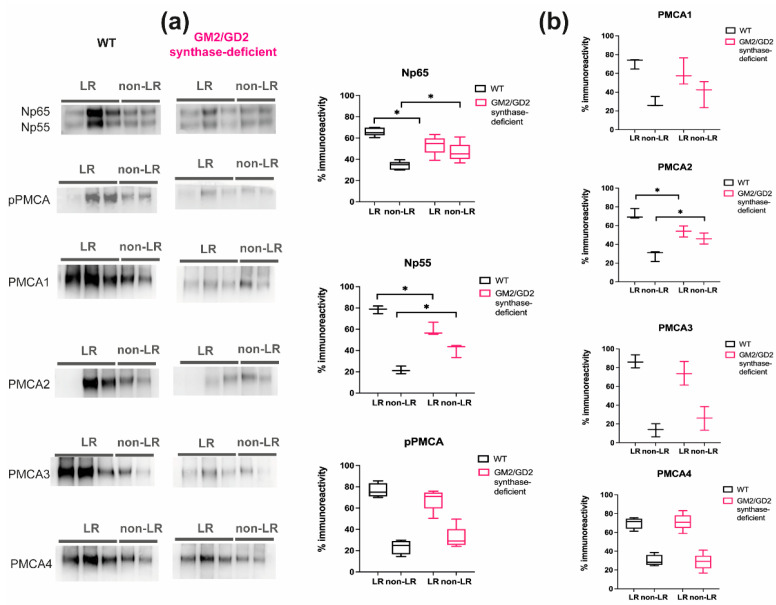
(**a**) Representative Western blots showing Neuroplastin 65 (Np65), Neuroplastin 55 (Np55) and PMCAs distribution in lipid raft (LR) and non-LR fractions isolated from cortical tissue of WT and GM2/GD2 synthase-deficient mice. (**b**) Box and whisker plots representing the percentage of immunoreactivity intensity in LR and non-LR fractions calculated using ImageJ. (* *p* < 0.05, Student’s *t*-test). WT = wild-type mice; pPMCA = pan PMCA encompassing all PMCA isoforms.

Figure 1 shows the lipid raft and the bulk membrane distribution of Neuroplastin 55, Neuroplastin 65 and PMCAs in WT and GM2/GD2 synthase-deficient mice cortices. In WT mice, the distribution of both Neuroplastin isoforms differs significantly between LR (Np65 68%; Np55 78%) and non-LR (Np65 32%; Np55 22%; *p* < 0.05, Student’s *t*-test and multiple t test; Supplementary Tables S1 and S2). In mice lacking complex gangliosides, Np65 is less enriched in LR (54%) and more dispersed in the bulk membrane (46%) so that the difference in LR and nLR localization is no longer significantly different (Supplementary Table S2). The situation is similar for Np55 and the difference between LR immunolocalization (59%) and non-LR immunolocalization (41%) in GM2/GD2 synthase-deficient mice is not significantly different. Therefore, in WT cortex, Neuroplastin is significantly more abundant in lipid rafts, whereas in mice lacking complex gangliosides, Neuroplastin is more dispersed throughout the membrane. In addition, the ratio of LR-associated Np65 and Np55 and the bulk membrane associated Np65 and Np55 (Supplementary Table S2) is significantly different between WT and GM2/GD2 synthase-deficient mice (2.1 vs. 1.3 LR/nLR ratio for Np65 and 3.7 compared to 1.5 ratio for Np55).

PMCA distribution in LRs and non-LRs was evaluated by using several antibodies with different specificities (Figure 1; Supplementary Tables S1 and S2). pPMCA antibodies recognize all 4 PMCA isoforms and can detect an overall disturbance in PMCA positioning, not attributing it to a specific isoform. According to the pPMCA antibody immunoreactivity, the distribution of total PMCA in LRs is similar to that of Neuroplastin: more pPMCA is present in LRs in WT mice (76%) compared to GM2/GD2 synthase-deficient animals (68%). For pPMCA, there is also an obvious dispersal from LRs to the bulk membrane in GM2/GD2 synthase-deficient mice. Using antibodies specific for individual PMCA isoforms, the situation is different. Concerning the distribution of PMCA isoforms in WT, PMCA1, PMCA2, and PMCA4 show similar immunoreactivity in lipid rafts: 71%, 72% and 70%, respectively. PMCA3 is even more concentrated in the lipid rafts in WT mouse with 86% of the total immunoreactivity. In GM2/GD2 synthase-deficient mice, the enrichment of total PMCA and the individual isoforms in LR are similar to WT. However, except for PMCA4, the immunoreactivity in lipid rafts, overall, is lower and dispersal from rafts to bulk membrane is detected similar to the altered Np dispersion pattern. The difference in lipid raft presence is most prominent and statistically significant for PMCA2 (72% immunoreactivity in WT mice compared to 54% in GM2/GD2 synthase-deficient animals). All PMCA isoforms, when regarded individually or combined, show preferential localization to LR compared to non-LR fraction. However, this distribution preference is reduced for PMCA1 and lost for PMCA2 in GM2/GD2 synthase-deficient mice. The differences in the ratios of LR/nLR immunoreactivity between WT and GM2/GD2 synthase-deficient mice are statistically significant for Neuroplastins and PMCA2, thus confirming the significant redistribution of these isoforms in GM2/GD2 synthase-deficient mice.

To confirm that the redistributions we detected for Neuroplastins and PMCAs are specific and do not result from a general disturbance of the membrane due to altered ganglioside composition, we evaluated the distribution of the LR marker flotillin 1 (Flot1) and the bulk non-LR marker transferrin receptor (TfR) in WT and GM2/GD2 synthase-deficient mice (KO) (Supplementary Figure S1). Both Flot1 and TfR are similarly distributed in WT and KO mice. Flot1 is localized mostly in rafts (78% and 81% for WT and KO mice, respectively). TfR distribution is concentrated in the bulk membrane fractions (75% for both WT and KO mice). These distributions are in concordance with the literature and confirm the successful lipid raft isolation [28,29,30].

### 2.2. Neuroplastin 65 Colocalizes with GM1 Ganglioside in Cultured Hippocampal Neurons

**Figure 2:**
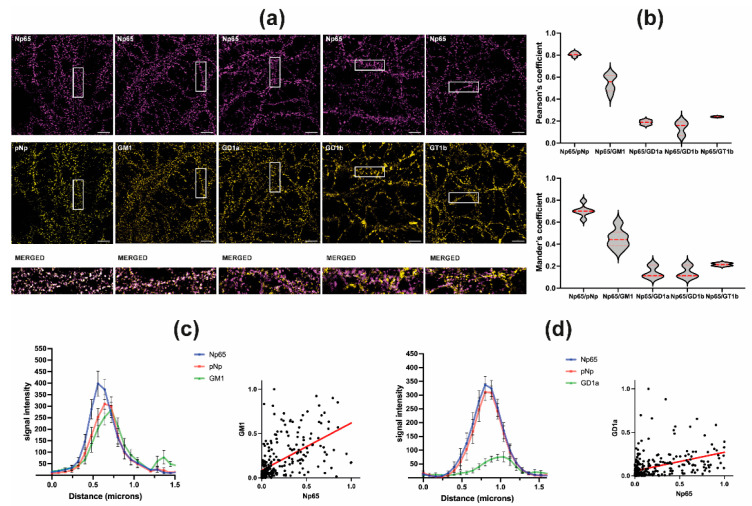
(**a**) Representative images of Np65, pNp, GM1, GD1a, GD1b, and GT1b immunofluorescence signals in cultured hippocampal neurons. Individual cultures were stained for Np65 (magenta, top row) and either pNp, GM1, GD1a, GD1b, and GT1b (yellow, middle row). The squares represent areas that were magnified, overlapped and shown as merged images in the bottom row (merged images of Np65 and pNp, GM1, GD1a, GD1b, and GT1b immunoreactivity, from left to right). Scale bar 5 microns. (**b**) Upper graph: Pearson’s coefficients showing highest colocalization of Np65 with pNp and GM1 and less with GT1b, GD1a, and GD1b. Lower graph: Mander’s coefficients showing highest colocalization of Np65 with pNp and GM1 and less with GT1b, GD1a, and GD1b. (**c**) Left: distribution of signal intensity of Np65, pNp, and GM1 over distance. Right: Correlation between Np65 and GM1 signal intensity. (**d**) Left: distribution of signal intensity of Np65, pNp, and GD1a over distance. Right: Correlation between Np65 and GD1a signal intensity.

After demonstrating a shift in submembrane localization of Nps and specific PMCA isoforms in mice lacking complex gangliosides, we wanted to ascertain if there is a preference of Np for a vicinity of a particular ganglioside. For that purpose, we performed colocalization studies of Neuroplastin with complex gangliosides in living neurons (Figure 2).

Np65-specific antibodies and pNp antibodies, that recognize both isoforms Np55 and Np65, were used to detect Neuroplastin expression and localization in cultured hippocampal neurons. Colocalization was analyzed by calculation of Pearson’s and Mander’s correlation coefficients. For Np65 and pNp, the highest Pearson’s (0.808) and Mander’s (0.703) coefficients were determined. When we compared colocalization of Np65 with complex gangliosides, we observed highest correlation of Np65 with GM1 in mature hippocampal neurons (22–39 days) with Pearson’s and Mander’s correlation coefficients for Np65/GM1 of 0.546 and 0.450, respectively. The other gangliosides showed much less overlap of immunoreactivity signals with Np65. Pearson’s and Mander’s coefficients for Np65/GD1a, Np65/GD1b and Np65/GT1b are 0.188 and 0.186, 0.142 and 0.142, 0.240 and 0.216, respectively (Figure 2a,b).

We analyzed the colocalization of Np65 with GM1 and GD1a in more detail. The distribution of Np65, pNp, and GM1 signal intensity over distance shows overlapping peak intensities of Neuroplastins and GM1, which suggests their close vicinity within the neuronal membrane (Figure 2c left). The normalized signal intensity distribution of GM1 and Np65 shows a correlation (r = 0.7554, Spearman’s correlation; R^2^ = 0.3262, F = 134.6, *p* < 0.0001, simple linear regression (Figure 2c right). The analysis of Np65/GD1a signal intensity over distance shows separate intensity peaks (Figure 2d, left). The normalized signal intensity distribution of GD1a and Np65 intensities shows only moderate correlation (r = 0.6517, Spearman’s correlation; R^2^ = 0.1784, F = 67.97, *p* < 0.0001, simple linear regression (Figure 2d right).

### 2.3. Antibody Engagement of GM1 Ganglioside Results in Prolonged Calcium Level Restoration

**Figure 3:**
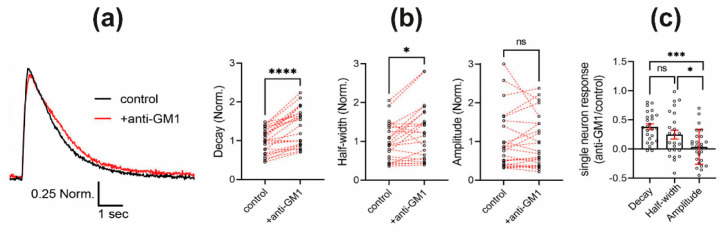
(**a**) Representative traces of electrically evoked somatic calcium transients before (control, black trace) and after 5 min treatment with anti-GM1 antibodies (+anti-GM1, red trace). (**b**) Decay time, half-width, and amplitude of the calcium transients were quantified, normalized, and plotted for each neuronal soma. Paired responses are connected by a segmented red line. For decay **** *p* < 0.0001 and half-width * *p* < 0.05 when paired control and +anti-GM1 are compared using Wilcoxon matched-pairs signed rank test (*n* = 24 neurons from 3 independent cultures). (**c**) +anti-GM1/ control ratio is shown for each single neuron and mean and SEM are displayed for each parameter. For decay time vs. amplitude *** *p* < 0.001 and half-width vs. amplitude * *p* < 0.05; ns = not significant.

After observing that altered ganglioside composition in GM2/GD2 synthase-deficient mice results in redistribution of Neuroplastin-PMCA in isolated brain lipid rafts (Figure 1) and that out of the four most abundant gangliosides in the brain, GM1 displayed very high co-localization with Neuroplastin in living hippocampal neurons (Figure 2), we investigated whether GM1 plays a role in calcium regulation through Neuroplastin-PMCA complexes in living hippocampal neurons. Therefore, we applied a monoclonal antibody against GM1 to acutely disturb GM1 interactions in Fluo-4-loaded living hippocampal neurons (Figure 3, Supplementary Videos S1 and S2). Traces of electrically evoked somatic calcium transients were recorded before (black trace in Figure 3a) and 5 min after application of anti-GM1 antibodies (+anti-GM1; red trace in Figure 3a). In particular, restoration to baseline levels after stimulus induced calcium increase was slower in the presence of anti-GM1 antibodies (Figure 3a). Indeed, the decay time and the half-width of individual somatic calcium transients were significantly increased by anti-GM1 antibodies (Figure 3b) resulting in changes as large as 49% and 25%, respectively (Figure 3c). Interestingly, the amplitude of the calcium transients was unaffected by anti-GM1 antibodies (Figure 3b,c), indicating a specific effect of the GM1 antibodies on the restoration of calcium levels. This effect is compatible with a decreased PMCA activity.

### 2.4. Brain Ganglioside Content and Composition Are Not Significantly Affected by Neuroplastin Deficiency

**Figure 4:**
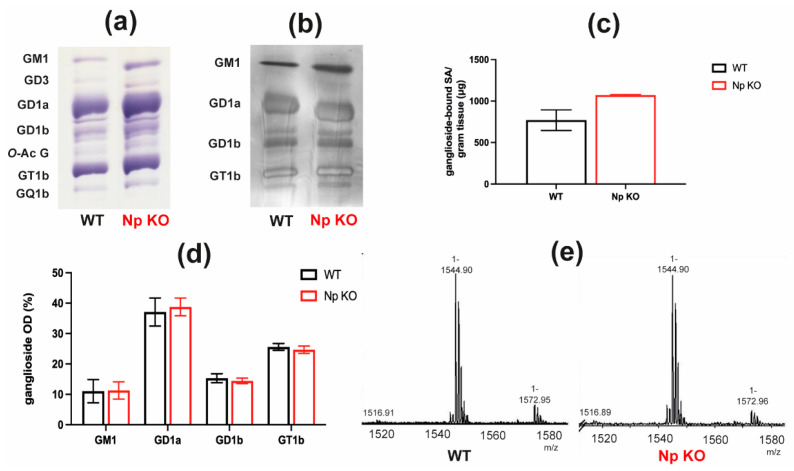
Ganglioside expression and composition in cortices of Neuroplastin-deficient (Np KO) compared to wild-type (WT) mice. (**a**) Representative high performance thin layer chromatography (HPTLC) plate showing no significant difference in ganglioside composition between cortices of WT and Np KO mice. (**b**) Representative cholera toxin subunit B (CTB) overlay following sialidase treatment showing no significant difference in ganglioside composition between WT and Np KO cortices. (**c**) Total ganglioside concentrations (μg ganglioside-bound sialic acids (SA) per gram fresh tissue) in cortices of WT and Np KO mice. (**d**) Quantification results of major ganglioside fractions separated by HPTLC, expressed as their relative proportion (%) of the total ganglioside content in the analyzed sample. (**e**) Mass spectra of GM1 ganglioside from WT and Np KO mouse cortices with major molecular ion marked.

To estimate the directionality of the Neuroplastin-PMCA-gangliosides link and determine whether Neuroplastin expression affects ganglioside expression and/or composition, we analyzed ganglioside expression and composition in cortical tissue from Neuroplastin-deficient mice (Np KO) using high performance thin layer chromatography (HPTLC), cholera toxin B (CTB) overlay analysis, quantification of ganglioside-bound sialic acids and mass spectrometry (MS) (Figure 4, Supplementary Figure S2 and Table S3).

The analysis of ganglioside content after separation by HPTLC followed by resorcinol stain visualization revealed no difference in ganglioside composition between WT and Np KO animals (Figure 4a), even though the overall intensity (total intensity of all bands) was higher in Np KO compared to WT mice. The relative quantification of individual ganglioside species (Figure 4d) is in line with the observed pattern on HPTLC and no significant differences in the proportions of gangliosides between WT and Np KO mice was observed. The same result was confirmed by CTB overlay analysis (Figure 4b) where we observed the same ganglioside composition pattern with apparent higher overall intensity of the staining in Np KO animals.

Figure 4c shows the quantification of ganglioside-bound sialic acids in Np KO mice compared to WT mice. While average total ganglioside-bound sialic acid concentration is higher in cortices of Np KO compared to WT mice, it was not statistically significant.

Figure 4e shows the representative mass spectra of GM1 ganglioside analyzed from Np KO animals compared to WT mice. No difference between the GM1 structure in WT compared to Np KO animals was detected. Further details of the mass spectrometry analysis are shown in Supplementary Figure S2. Structural characterization by electrospray ionization (ESI) MS identified major ganglioside species typical for mouse brains. These include GT1 and *O*-acetylated (*O*-Ac-) GT1, GQ1 and *O*-Ac-GQ1, GD1, *O*-Ac-GD1 and GalNAc-GD1, and GM1. All these ganglioside species are detected in both WT and Np KO mice without apparent differences between WT and Np KO at the structural level. Further details regarding the ganglioside structures assigned to detected *m*/*z* values and molecular ion types are presented in Supplementary Table S3. Furthermore, no difference between the ceramide composition (i.e., fatty acid length and saturation level present in the ganglioside ceramide core) in WT and Np KO mice was detected (Supplementary Table S3). The assignments are consistent with structures previously identified in mouse brains [31].

## 3. Discussion

**Figure 5:**
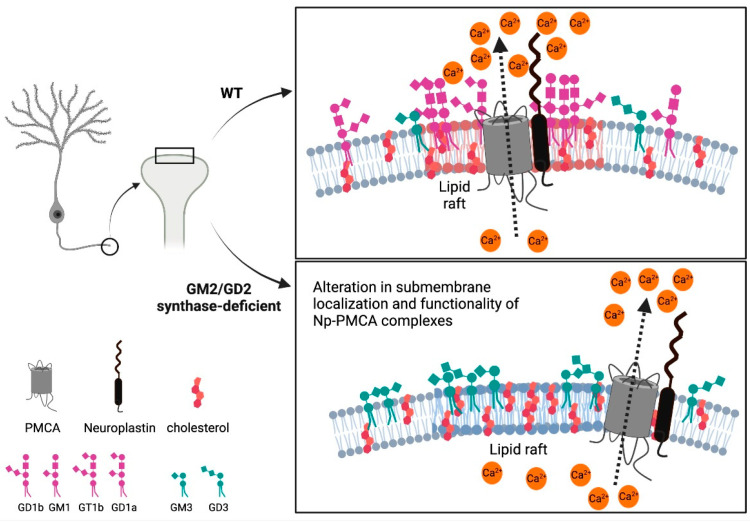
Schematic representation of the consequences of altered ganglioside composition on the submembrane localization of PMCA-Neuroplastin complexes, PMCA function and calcium regulation.

This work highlights the importance of gangliosides, particularly GM1, in providing the molecular environment for PMCA-Neuroplastin complexes. We show that by disrupting complex ganglioside biosynthesis and thus affecting their composition in the membrane as well as removing GM1 ganglioside, the submembrane localization of Neuroplastin in complex with specific PMCA isoforms is changed and alters the calcium regulation (Figure 5).

Gangliosides, the most complex glycosphingolipids, are known to modulate ion homeostasis, including calcium signaling. However, the reports regarding their exact role are often conflicting and do not offer a mechanistic explanation of the observed phenomena. One possible explanation is that gangliosides seem to bind calmodulin and may thereby affect the activity of calmodulin-dependent enzymes such as PMCA [32,33]. Poly-sialogangliosides appear to have stimulatory effects on PMCA, while mono-sialogangliosides and asialo-gangliosides have either an inhibitory effect or no effect [34,35,36], indicating that the complexity of ganglioside structure, as well as the presence of sialic acid residues, fine-tunes the PMCA activity. Our approach was to first examine the submembrane localization of PMCAs and Neuroplastins in a genetically engineered mouse model that lacks the GM2/GD2 synthase enzyme. The phenotype of GM2/GD2 synthase-deficient mice includes motor neuropathy, affecting behavior and coordination, axon degeneration, dysmyelination and deficits in cognitive function [37,38,39,40,41,42]. In recent years, congenital mutations in the human *B4GALNT1* gene encoding GM2/GD2 synthase have been associated with the human autosomal recessive disorder, hereditary spastic paraplegia (HSP) [43,44,45,46,47]. HSP symptoms include lower extremity spasticity and muscle weakness causing abnormal gait, intellectual disability, dysarthria, peripheral neuropathy, and extrapyramidal and cerebellar deficits. Even though HSP is undoubtedly attributed to loss of GM2/GD2 synthase, the path leading from deficient ganglioside synthesis to these complex symptoms is veiled in mystery. Therefore, it is particularly important to elucidate the molecular events from alteration in ganglioside composition of the membrane to the complex disease manifestation. Interestingly, a missense mutation in *ATP2B4*, encoding PMCA4, is also associated with one form of familial spastic paraplegia and calcium dysregulation was proposed to be associated with the pathogenesis of this disease [48,49].

Since mouse and human complex brain gangliosides are similar in both structure and relative expression, the mouse model we used can be considered a phenocopy of the human disease. We show the dissipation of both Neuroplastin isoforms from organized lipid rafts to the bulk membrane (Figure 1) evident from the abundance of individual proteins in LRs and the bulk membrane (Supplementary Table S1), as well as calculated immunoreactivity ratios for LR/nLR (Supplementary Table S2) However, although the vast majority of PMCA is associated with Neuroplastin [6,7,8], not all PMCA isoforms show the same distribution pattern as Neuroplastin (Figure 1). Specifically, the PMCA4 isoform appears to be less sensitive to disruption of the ganglioside environment and Neuroplastin repositioning, while all other PMCA isoforms follow the Neuroplastin dispersion pattern (Figure 1 and Supplementary Tables S1 and S2). On the other hand, PMCA2 isoform distribution resembles the Np distribution most closely, both in immunoreactivity signal in lipid rafts, as well as calculated immunoreactivity ratio for LR/nLR. The notion that PMCA activity is partially regulated by the appropriate lipid composition of membrane lipid rafts is supported by other research showing the elevation of GM1 and loss of PMCA activity after alteration of the lipid raft composition by cholesterol depletion in primary neurons [25]. Furthermore, sialic acid residues in gangliosides interact with Ca^2+^ ions via electrostatic interactions. The controlled binding and release of Ca^2+^ ions by negatively charged sialic acid residues on gangliosides plays an important role in neurotransmission, thereby highlighting an additional dimension of the function of gangliosides in calcium homeostasis [50,51,52]. Research on malfunctions of PMCA due to mutations suggest that the disease phenotypes are related to impaired calcium modulation in submembrane microdomains, which leads to the defective control of PMCA activity due to important dependent interactors which reside in membrane microdomains [49,53]. We found that specific gangliosides are indeed interactors necessary for ensuring optimum PMCA-Np interactions and positioning. Considering the role of gangliosides in long-term potentiation (LTP) as a potential molecular mechanism explaining memory formation through Ca^2+^ ions interactions [51] and that PMCA2 KO animals show the most severe neuroplasticity problems among PMCA isoform KO animals [54], it is especially riveting that PMCA2 is the most affected isoform concerning redistribution from lipid rafts in our mouse model (Figure 1, Supplementary Tables S1 and S2).

Colocalization data in living neurons (Figure 2) showed that out of the four most abundant gangliosides in the brain, Np prefers the vicinity of GM1. By extension, PMCA positioning and therefore function depends on ganglioside composition. Since Neuroplastin is obviously sensitive to the presence and/or absence of specific gangliosides, and different gangliosides populate different lipid rafts [55], this could also explain the differential effect of poly-sialogangliosides and mono-sialogangliosides on PMCA activity [34,35]. Due to technical reasons, colocalization analysis for gangliosides and PMCA was not performed in the same system. Specifically, the PMCA epitopes recognized by antibodies are positioned inside the membrane and require membrane permeabilization for unmasking. As we performed the staining in living cells, detergents permeabilizing the membrane could not be used.

Having observed that genetically modified ganglioside composition results in altered levels and distribution of Neuroplastin-PMCA in isolated brain rafts (Figure 1) and that GM1 displayed very high colocalization with Neuroplastin in intact hippocampal neurons (Figure 2), we wondered whether GM1 plays a role in calcium regulation mediated by Neuroplastin-PMCA complexes in living hippocampal neurons. There are reports linking GM1 to calcium homeostasis through TrkA receptor stimulation and consequent opening of calcium channels [56], and, in general, GM1 is often in the spotlight more than other gangliosides, especially its implication in pathology of different neurodegenerative disorders [52,57,58]. By blocking GM1 with a monoclonal antibody and therefore disrupting its interactions with PMCA-Np, we observed a significant difference in the decay time and half-width of individual somatic calcium transients in living neurons (Figure 3 and Supplementary movies S1 and S2). However, the amplitude of the calcium transients was unaffected (Figure 3), indicating a rather specific effect of the GM1 antibody on calcium extrusion which is compatible with a reduced PMCA activity, as was also observed in Neuroplastin-deficient neurons [13]. Therefore, our results suggest that the intact GM1 environment is important for Neuroplastin-PMCA complex function in calcium regulation.

The intricacy of the ganglioside-Neuroplastin relationship is additionally highlighted when we examine the results of ganglioside analysis in Np KO brains (Figure 4, Supplementary Figure S2 and Table S3). There is no significant compositional or structural change detected in animals lacking Neuroplastin, only a slight overall higher ganglioside-bound sialic acid content. The dramatic changes we see in Neuroplastin expression and localization in the brains of ganglioside deficient mice [21], compared to no or very subtle changes in ganglioside content we see in Neuroplastin deficient mice, demonstrates the directionality of that relationship, where the lack of gangliosides influences Neuroplastin expression, but not vice versa.

One function assigned to complex gangliosides is the maintenance of calcium homeostasis during neurodevelopment and aging, which is impaired in disorders linked to altered ganglioside composition [59]. Furthermore, a disarranged ganglioside environment of Neuroplastin with aging [60] and altered lipid raft functionality during neurodegeneration [61] were reported. In addition, gangliosides are reported to influence cholesterol homeostasis [62], which could be a contributing factor in PMCA regulation [63]. It appears that reshaping the ganglioside milieu of neuronal membranes causes a domino effect, leading to functional changes in ion regulation, and probably other cellular systems as well. Considering the growing body of evidence linking aberrant ganglioside metabolism to human disorders [23,43,44,45,46,47], without an adequate explanation regarding the mechanism leading from altered ganglioside composition of the membrane to the clinical presentation of the disease, our study will facilitate an understanding of the etiopathology of these complex disorders, as well as hopefully contribute to therapy development.

## 4. Materials and Methods

### 4.1. Animals

A total of 19 adult male mice, aged 2–6 months (mean age 4.5 months, median age 6 months) were used for this study (10 wild-type (WT), 6 *B4galnt1 null* and 3 Neuroplastin-deficient (KO) mice). Wild-type littermates were used as controls. All mice had the same genetic background (C57BL/6). *B4galnt1 null* animals, lacking the enzyme GM2/GD2 synthase (UDP-*N*-acetyl-D-galactosamine: GM3/GD3 *N*-acetyl-D-galactosaminyltransferase; EC 2.4.1.92), were previously characterized and their genotype confirmed by genotyping [37]. Instead of expressing complex gangliosides, simpler gangliosides GM3 and GD3 are accumulated in these animals [37,38,39,40,41,42]. Neuroplastin-deficient mice, lacking the Neuroplastin protein, were previously characterized [17] and their genotype was confirmed by genotyping.

The animals were group-housed, kept under standardized temperature and humidity and a 12 h light-dark cycle with water and food ad libitum in standard cages. For all experiments, the mice were anesthetized with an isoflurane (Vetopharma Animal Health, S.L., Barcelona, Spain) overdose until loss of consciousness and then decapitated. Brains were rapidly removed, dissected, and kept at −80 °C until use. All experimental procedures were performed in accordance with the ARRIVE guidelines. All procedures were approved by regional ethics committees for scientific experiments and approved by the appropriate institutions in accordance with institutional and government guidelines (please see Institutional Review Board Statement for details).

### 4.2. Lipid Raft Isolation

Lipid rafts were isolated as previously described by discontinuous sucrose gradients ultracentrifugation [28,29,64]. Briefly, 70 ± 5 mg of brain cortical tissue was homogenized, the nuclear fraction removed, and the cell membrane pellet obtained by centrifugation (100,000× *g* at +4 °C, 30 min; ultracentrifuge Beckman Optima XL-80 K, Beckman Coulter, Inc., Brea, CA, USA). The pellet was homogenized in a buffer containing the non-ionic detergent Brij O20 (Sigma-Aldrich, St. Louis, MO, USA) and ultracentrifuged (140,000× *g*, at +4 °C, 18 h) in a discontinuous sucrose (Kemika, Zagreb, Croatia) gradient which consisted of 85% sucrose mixed with sample in 1:1 ratio, overlaid with 35% sucrose solution followed by 3% sucrose solution (ultracentrifuge Beckman Optima XL-80 K, Beckman Coulter, Inc., Brea, CA, USA). After centrifugation, fractions were collected top to bottom and analyzed by Western blotting.

### 4.3. Western Blotting

Western blotting was performed as previously described [21,28] in order to assess the lipid raft and the bulk membrane distribution of proteins of interest. Briefly, equal volumes of isolated lipid raft fractions were loaded onto precast 4–12% Bis Tris gels (Thermo Fisher Scientific, Life Technologies Corporation, Carlsbad, CA, USA) and resolved in MOPS running buffer (Thermo Fisher Scientific, Life Technologies Corporation, Carlsbad, CA, USA). Equal loading volumes opposed to equal protein concentrations were used to allow direct comparisons of protein distribution across fractions [28,29,30]. In order to check the amount of protein loaded and to ensure successful transfer of proteins onto the PVDF membrane (Thermo Scientific, Rockford, IL, USA), we used Stain-free technology [65]. After transfer and before blocking step, membranes were incubated with No-Stain protein Labeling reagent (Thermo Fisher, Life Technologies Corporation, Carlsbad, CA, USA) for 10 min, rinsed three times in distilled water and membranes were visualized on BioRad Chemidoc MP System (Bio-Rad Laboratories, Inc., Hercules, CA, USA). Additionally, to ensure that the immunoreactivity pattern detected on Western blotting is indeed a result of the membrane redistribution of the protein of interest and not an artefact, the protein concentration of all collected fractions after lipid raft isolation for WT and KO mice was determined according to Bradford (Supplementary Figure S3). After separation and electro-transfer onto PVDF membrane, the membranes were incubated in a blocking reagent and then with primary antibody overnight, followed by the appropriate secondary antibody. The antibody details are given in Table 1. Protein bands were visualized using ECL Western blotting substrate (Bio-Rad Laboratories, Inc., Hercules, CA, USA) and imaged on BioRad Chemidoc MP System. The bands were quantified using ImageJ analysis software (1.53i version, NIH, Bethesda, MD, USA).

### 4.4. Rat Neuronal Culture Preparation

Neuronal cultures were prepared as established [15]. The hippocampi were dissected from rat embryos on embryonic day 18 (E18) and transferred to a 15 mL centrifuge tube containing ice-cold HBSS solution (Hanks’ Balanced Salt Solution with phenol red, without calcium and magnesium, Merck KGaA, Darmstadt, Germany). The tissue was trypsinized by the addition of 1 mL Trypsin (Merck KGaA, Darmstadt, Germany) and incubation at 37 °C for 8 min. After incubation, the trypsin solution was removed, and the hippocampal tissue was washed twice with 2 mL DMEM (Dulbecco’s Modified Eagle Medium, Gibco, Waltham, MA, USA) with 10% horse serum, penicillin, streptomycin and glutamine (all from Gibco, Waltham, MA, USA), and further dissociated by pipetting approximately 5 times through a flame-polished Pasteur pipette. Dissociated neurons were transferred to a fresh centrifuge tube and the number of neurons was counted using a hemocytometer (Merck KGaA, Darmstadt, Germany). 50,000 cells were plated on poly-D-lysine coated coverslips of 12-well plates. After incubation for 2 h, the media replaced with 1 mL Neurobasal supplemented with B27 per well (all from Gibco, Waltham, MA, USA). The neurons were then allowed to grow in a 37 °C and 5% CO_2_ incubator.

### 4.5. Live Neuron Staining

Immunofluorescence of live neurons was performed as follows: primary antibodies were added to medium used for incubating neurons (1% horse serum, 1% glutamine in Neurobasal medium, all from Gibco, Waltham, MA, USA). For dilutions, see Table 1. After incubation for 20 min at 37 °C, neurons were washed with medium. Secondary fluorescent antibodies were then added to the incubation medium, and neurons were incubated for 20 min at 37 °C. For dilutions, see Table 1. After washing, neurons were fixed using 4% PFA (Sigma-Aldrich, St. Louis, MO, USA) for 8 min at 37 °C. Neurons were then washed in PBS, incubated with DAPI (Sigma-Aldrich, St. Louis, MO, USA) for 10 min, washed again and mounted using Mowiol mounting medium (Sigma-Aldrich, St. Louis, MO, USA)**.**

### 4.6. Confocal Imaging and Image Analysis

Primary neuronal cultures were imaged using Leica TCS SP5 Confocal microscope (Leica Microsystems, Wetzlar, Germany). Images were deconvolved and colocalization was analyzed using ImageJ software (1.53i version, NIH, Bethesda, MD, USA) with Diffraction PSF 3D and JACoP plugins [67]. Intensities and distribution of intensities over distance were analyzed using plot profile.

### 4.7. Single Neuron Calcium Imaging Using Fluo4-AM

Mature hippocampal neurons (20–22 DIV) were incubated with 1 µL Fluo-4 AM (Thermo Fisher, Life Technologies Corporation, Carlsbad, CA, USA) for 30 min. The neurons were transferred to a RC-49MFSH magnetic imaging/recording chamber with removable electrodes (Warner Instruments, Hamden, CT, USA) and covered with 900 μL of 1× Tyrodes buffer (119 mM NaCl, 2.5 mM KCl, 25 mM HEPES, 30 mM Glucose, 2 mM MgCl_2_ and 2 mM CaCl_2_, all from Sigma-Aldrich, St. Louis, MO, USA) at 32 °C. An inverted microscope (Observer. D1; Zeiss, Oberkochen, Germany) equipped with a 63×/1.2 N.A. objective, GFP/RFP single band exciters ET filter set (excitation 470/40, excitation 572/35, emission 590/22, dichroic 59022BS), and an EMCCD camera (Evolve 512 Delta; Photometrics, Tucson, AZ, USA) controlled by VisiView^®^ Software (Visitron Systems GmbH, Puchheim, Germany) was used to perform live imaging (33 Hz acquisition rate). For each experiment, the somatic calcium transients were evoked electrically by extracellular depolarization using 5 pulses (1 ms duration each) at 20 Hz at 32 °C. Somatic calcium transient was obtained in basal condition (control) for each neuron. After 2 min of resting, neurons were incubated with a purified IgG monoclonal anti-GM1 antibody (1 μg/mL; [66,68]) dissolved in 1× Tyrodes buffer (Sigma-Aldrich, St. Louis, MO, USA) for 5 min and stimulated again.

### 4.8. Ganglioside Purification

Ganglioside extraction procedure was performed as previously described [31,69,70]. Briefly, dissected brain regions were homogenized in ice-cold distilled water (W) in a Potter–Elvehjem glass-Teflon homogenizer (DeOtto Lab, Zagreb, Croatia). Lipids were extracted using organic solvents chloroform (C):methanol (M) (1:2, by vol.), followed by partition and repartition by adding M and W to a final volume ratio 1:1:0.7 (chloroform was from T.T.T., Sveta Nedjelja, Croatia; methanol from Honeywell Riedel-de Haen, Seelze, Germany). Upper phases were collected, evaporated to dryness and further purified by gel filtration Sephadex-G25 (Sigma-Aldrich, St. Louis, MO, USA) [71].

### 4.9. Ganglioside Analyses

Quantitative analysis of ganglioside-bound sialic acid content was determined spectrophotometrically as previously described [31,72]. The absorbances of samples and *N*-acetylneuraminic acid (Sigma-Aldrich, St. Louis, MO, USA) used as a standard in a range of known concentrations were determined at 580 nm. The contents of ganglioside-bound sialic acids are expressed as microgram of ganglioside-bound sialic acids per gram of fresh tissue w.w. The purified samples were qualitatively analyzed by HPTLC, CTB overlay and mass spectrometry. HPTLC separation of individual ganglioside species was performed as previously described [28,31,73]. Purified samples were dissolved in C:M:W (60:30:4.5, by vol.) and the aliquots spotted to the HPTLC plate (Merck KGaA, Darmstadt, Germany). Resolved gangliosides were detected by resorcinol–HCl reagent [72]. Densitometry was performed using Image Lab software (Bio-Rad Laboratories, Inc., Hercules, CA, USA) and bands quantified using ImageJ analysis software (1.53i version, NIH, Bethesda, MD, USA) as percentage in immunoreactivity intensity. Relative quantification of individual ganglioside species is expressed as their relative proportions (%) in total ganglioside content in the analyzed sample. For CTB overlay assay the plates were developed in the same way and CTB overlay performed as previously described [28,74]. The plates were immersed in 0.3% poly(isobutyl methacrylate) (Sigma-Aldrich, St. Louis, MO, USA) in *n*-hexane (Honeywell Riedel-de Haen, Seelze, Germany), dried and overlayed with aqueous buffer containing *V. cholerae* sialidase diluted to 30 mU/mL. After 3 h at 37 °C the plates were incubated in blocking reagent followed by incubation in HRP-conjugated CTB (Invitrogen, Life Technologies Corporation, Carlsbad, CA, USA; 1:20,000) for 1 h at ambient temperature. The bands were visualized using ECL Western blotting substrate (Bio-Rad Laboratories, Inc., Hercules, CA, USA) and imaged on BioRad Chemidoc MP System (Bio-Rad Laboratories, Inc., Hercules, CA, USA). Densitometry was performed using Image Lab software (Bio-Rad Laboratories, Inc., Hercules, CA, USA) and bands quantified using ImageJ analysis software (1.53i version, NIH, Bethesda, MD, USA). Mass spectrometry analysis was performed on a Bruker amaZon ETD ion trap system (Bruker Daltonik GmbH, Bremen, Germany) equipped with Apollo electrospray ionization source as previously described [31]. Purified gangliosides were dissolved in methanol at 1.6 μM concentrations and introduced into the electrospray ionization source by direct infusion. All spectra were acquired in negative ion mode as previously reported [31]. The MS data were extracted and analyzed (charge deconvolution and data reduction) using Bruker DataAnalysis software 4.0 (Bruker Daltonik GmbH, Bremen, Germany).

### 4.10. Statistics

All the graphs and statistical calculations were produced using GraphPad-Prism 9.2.0 (GraphPad Software, San Diego, CA, USA). The distribution of proteins in LR and non-LR is shown as mean and standard deviation (SD). Distribution between WT and KO were compared using unpaired t-tests, and the distribution of proteins in LRs and nLRs in one genotype was performed using a multiple *t* test and statistical significance determined using the Holm-Šídák method. If the *p*-value was lower than 0.05, the results were considered significant in all the cases. For culture stainings, correlations between ganglioside and protein intensity were tested using Spearmen’s coefficient. For calcium imaging, raw data outliers were removed using the ROUT method (Q = 1) and compared using a paired t test. If the *p*-value was lower than 0.05, the results were considered significant in all the cases. Normalized control calcium transients were compared to normalized calcium transients after anti-GM1 treatment using Wilcoxon matched pairs signed rank test and displayed as mean and standard error of mean (SEM).

## Figures and Tables

**Table 1:** List of primary and secondary antibodies used for live neuron staining (LNS) and Western blotting (WB).

Antibody	Host Species	Supplier	Cat. Number	Dilution
**Primary Antibodies**				
Anti-Transferrin receptor	Mouse	Thermo Fisher, Life Technologies Corporation, Carlsbad, CA, USA	136800	1:2000
Anti-Flotillin-1		BD Biosciences, Franklin Lakes, NJ, USA	610821	1:1000
Anti-Neuroplastin 65	Goat	R&D Systems, Minneapolis, MN, USA	AF5360	1:200 LNS 1:1000 WB
Anti-pan Neuroplastin	Sheep	R&D Systems, Minneapolis, MN, USA	AF7818	1:200 LNS 1:1000 WB
Anti-pan PMCA	Mouse	Abcam, Cambridge, UK	ab2825	1:500
Anti-PMCA4			ab2783	1:1000
Anti-PMCA1	Rabbit		ab190355	1:1000
Anti-PMCA2			ab3529	1:1000
Anti-PMCA3		Novus Biologicals, Bio-Techne Ltd., Abingdon, UK	NBP1-59465	1:1000
Anti-GM1 ganglioside	Mouse	Monoclonal antibodies prepared and validated as reported [66]		4.3 μg/mL
Anti-GD1a ganglioside				0.64 μg/mL
Anti-GD1b ganglioside				2 μg/mL
Anti-GT1b ganglioside				1.84 μg/mL
**Secondary Antibodies**				
Anti-goat Cy5	Donkey	Jackson ImmunoResearch Europe Ltd., Ely, UK	705-175-147	1:1000
Anti-sheep Cy3			713-165-003	1:1000
Anti-mouse 488			715-545-150	1:1000
Anti-mouse HRP			715-035-150	1:50,000
Anti-goat HRP			705-035-003	
Anti-sheep HRP			713-035-147	
Anti-rabbit HRP			711-035-152	

## Data Availability

The data is presented in manuscript Figures and Supplementary Materials. The raw data from this study are available on reasonable request from the corresponding author.

## Supplementary Materials

The following are available online at https://www.mdpi.com/article/10.3390/ijms222413590/s1.
