# Supplementary material for: Plasma Membrane Calcium ATPase-Neuroplastin Complexes Are Selectively Stabilized in GM1-Containing Lipid Rafts
Source: Int J Mol Sci. 2021 Dec 18;22(24):13590. doi: 10.3390/ijms222413590 (PMC8708829; doi:10.3390/ijms222413590)
Supplement: Supplementary file 1 [file ijms-22-13590-s001.zip › Ilic et al, Supplementary files/Ilic et al, PMCA-Np complexes in GM1-containing lipid rafts Supplementary material FIN.pdf]

Supplementary material for: Ilic et al, Plasma membrane calcium ATPase-Neuroplastin complexes are selectively stabilized in GM1-containing lipid rafts

Table S1. Data on Neuroplastins (Np55 and Np65) and PMCAs distribution in lipid raft (LR) and non-LR (bulk membrane) in WT and KO (GM2/GD2 synthase-deficient) animals. Data represent immunoreactivity intensity calculated using ImageJ. pPMCA = pan PMCA encompassing all PMCA isoforms. Statistically significant results (in red) are marked * ($p<0.05$, Student's t-test). SD = standard deviation.

	**WT (mean±SD)**	**KO (mean±SD)**
LR (%)		
**Np65**	**67.65±1.99***	**54.47±9.33***
**Np55**	**78.44±3.57***	**59.43±6.18***
pPMCA	76.42±6.7	67.94±10.14
PMCA1	71.09±5.61	60.98±14.24
**PMCA2**	**71.81±5.68***	**53.84±5.87***
PMCA3	86.46±6.94	73.93±12.59
PMCA4	70.05±6.07	71.31±8.68
Non-LR (%)		
**Np65**	**32.35±1.99***	**45.53±9.33***
**Np55**	**21.56±3.57***	**40.57±6.18***
pPMCA	23.58±6.7	32.06±10.14
PMCA1	28.91±5.61	39.02±14.24
**PMCA2**	**28.19±5.68***	**46.16±5.87***
PMCA3	13.54±6.94	26.09±12.59
PMCA4	29.95±6.07	28.69±8.68

Table S2. Data on Neuroplastins (Np55 and Np65) and PMCAs distribution in lipid raft (LR) and non-LR (bulk membrane) in WT and KO (GM2/GD2 synthase-deficient) animals. Data represent immunoreactivity intensity calculated using ImageJ. The comparison between LR-associated immunoreactivity and nLR-associated immunoreactivity in individual genotypes (e.g. immunoreactivity for Np65 in LR and non-LR for WTs) was performed using a multiple *t* test and statistical significance determined using the Holm-Šídák method. Immunoreactivity ratio LR/nLR represents the ratio of LR/nLR distributions of individual proteins compared between WT and KO mice (Student's t-test). pPMCA = pan PMCA encompassing all PMCA isoforms. Statistically significant results (in red) are marked * ($p<0.05$). SD = standard deviation.

		LR (%)	Non-LR (%)	Immunoreactivity ratio LR/nLR
**WT (mean±SD)**	**Np65**	**67.65±1.99***	**32.35±1.99***	**2.10±0.19***
	**Np55**	**78.44±3.57***	**21.56±3.57***	**3.72±0.77***
	**pPMCA**	**76.42±6.7***	**23.58±6.7***	3.58±1.63
	**PMCA1**	**71.09±5.61***	**28.91±5.61***	2.54±0.65
	**PMCA2**	**71.81±5.68***	**28.19±5.68***	**2.66±0.83***
	**PMCA3**	**86.46±6.94***	**13.54±6.94***	8.27±5.71
	**PMCA4**	**70.05±6.07***	**29.95±6.07***	2.43±0.62
**KO (mean±SD)**	Np65	54.47±9.33	45.53±9.33	**1.27±0.42***
	Np55	59.43±6.18	40.57±6.18	**1.51±0.42***
	**pPMCA**	**67.94±10.14***	**32.06±10.14***	2.32±0.81
	PMCA1	60.98±14.24	39.02±14.24	1.86±1.24
	PMCA2	53.84±5.87	46.16±5.87	**1.19±0.28***
	PMCA3	73.93±12.59	26.09±12.59	3.62±2.54
	**PMCA4**	**71.31±8.68***	**28.69±8.68***	2.68±1.20

Figure S1.

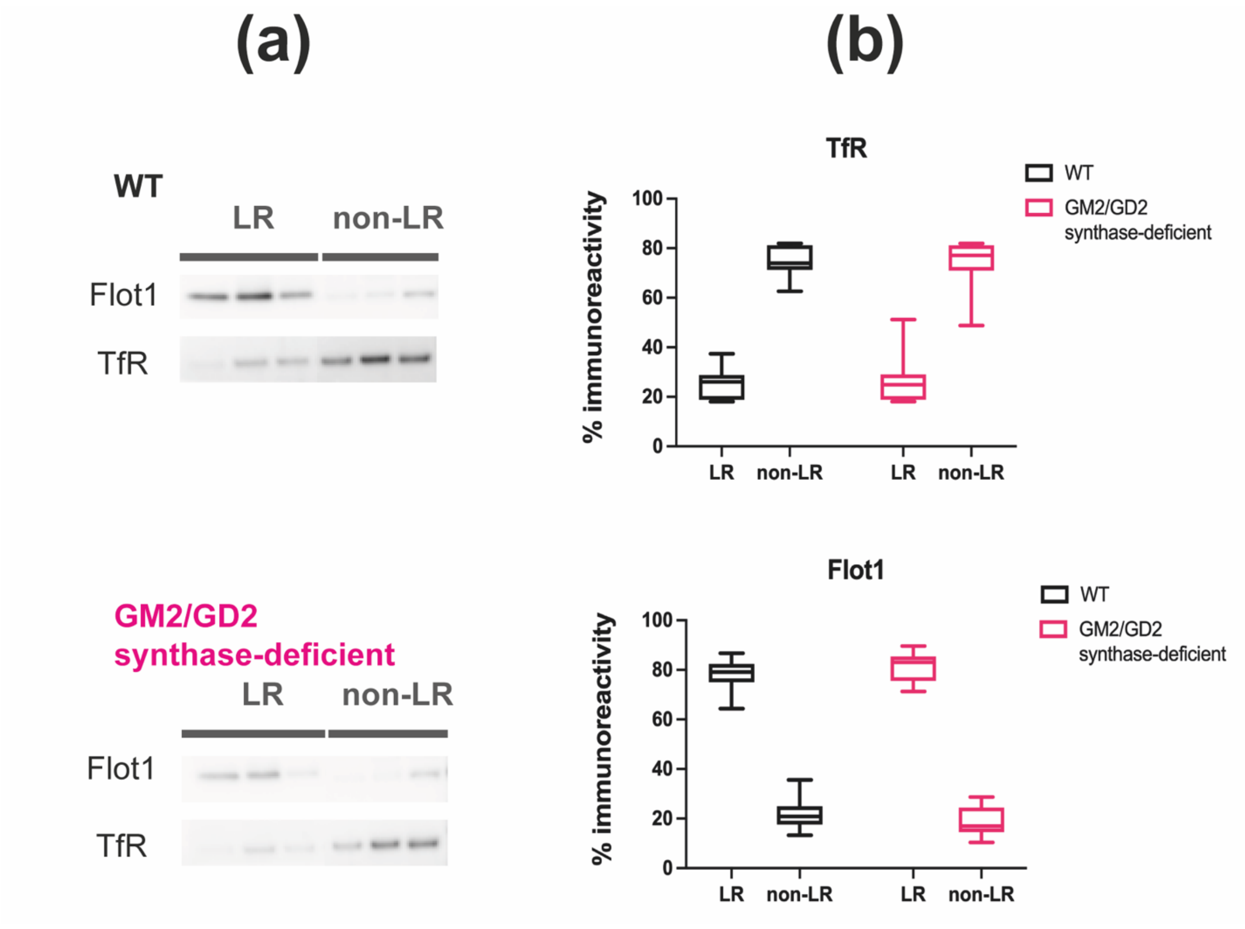

Figure S1. (a) Representative Western blots showing lipid raft (LR) marker flotillin 1 (Flot1) and non-lipid raft (non-LR) marker transferrin receptor (TfR) distribution in fractions following lipid raft isolation. (b) Box and whisker plots representing the percentage of immunoreactivity intensity in LR and non-LR fractions calculated using ImageJ. WT = wild-type mice.

Figure S2.

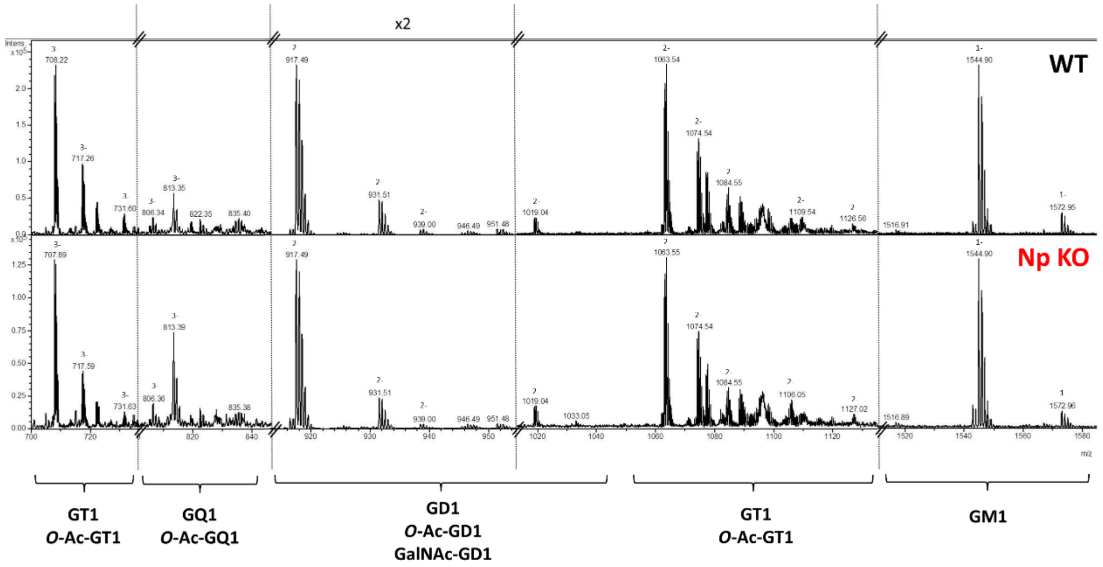

Figure S2. Mass spectra of total gangliosides from WT and Neuroplastin-deficient (Np KO) mouse cortices. Major molecular ions are indicated.

Table S3. Mass spectrometry data assigned to major ganglioside structures detected in cortices of wild-type (WT) and Neuroplastin-deficient (KO) mice.

**Molecular ion type**	**Detected *m/z***	**Assigned ganglioside structure**
$[M-3H^+]^{3-}$	708.55; 708.56<br>WT KO	GT1 (d18:1/18:0)
$[M-3H^+]^{3-}$	717.59	GT1 (d20:1/18:0)
$[M-3H^+]^{3-}$	721.92	*O*-Ac-GT1 (d18:1/18:0)
$[M-3H^+]^{3-}$	731.60; 731.63<br>WT KO	*O*-Ac-GT1 (d20:1/18:0)
$[M-3H^+]^{3-}$	805.45	GQ1 (d18:1/18:0)
$[M-3H^+]^{3-}$	828.52; 828.55<br>WT KO	*O*-Ac-GQ1 (d20:1/18:0)
$[M-2H^+]^{2-}$	917.49	GD1 (d18:1/18:0)
$[M-2H^+]^{2-}$	931.51	GD1 (d20:1/18:0)
$[M-2H^+]^{2-}$	938.50	*O*-Ac-GD1 (d18:1/18:0)
$[M-2H^+]^{2-}$	1019.04	GalNAc-GD1 (d18:1/18:0)
$[M-2H^+]^{2-}$	1033.05; KO<br>1033.08 WT	GalNAc-GD1 (d20:1/18:0)
$[M-2H^+]^{2-}$	1063.05	GT1 (d18:1/18:0)
$[MNa-3H^+]^{2-}$	1074.04	GT1 (d18:1/18:0)^{Na}
$[M-2H^+]^{2-}$	1077.06; WT<br>1077.07 KO	GT1 (d20:1/18:0)
$[M-2H^+]^{2-}$	1084.04; WT<br>1084.05 KO	*O*-Ac-GT1 (d18:1/18:0)
$[MNa-3H^+]^{2-}$	1095.02;<br>1095.03	*O*-Ac-GT1 (d18:1/18:0)^{Na}
$[MNa-3H^+]^{2-}$	1109.03	*O*-Ac-GT1 (d20:1/18:0)^{Na}
$[M-H^+]^{-}$	1544.90	GM1 (d18:1/18:0)
$[M-H^+]^{-}$	1572.95; WT<br>1572.96 KO	GM1 (d20:1/18:0)

Figure S3.

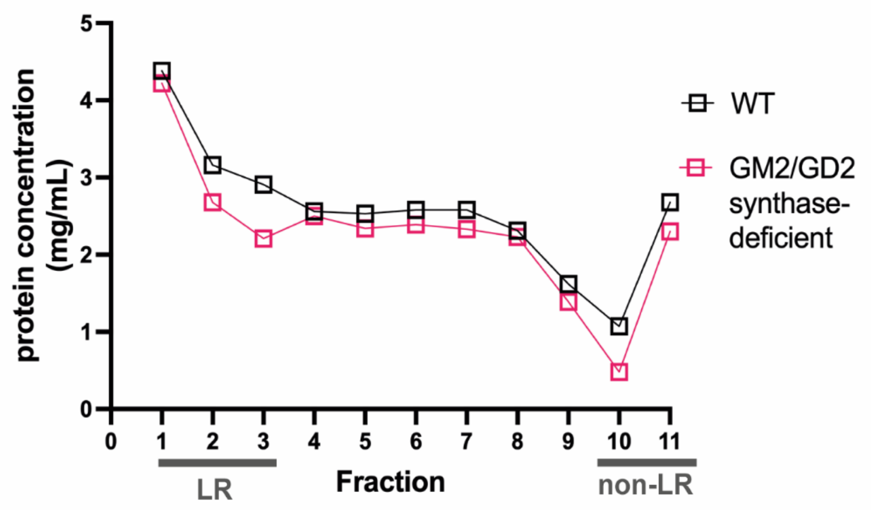

Figure S3. Concentration of total proteins across all fractions after lipid raft isolation determined using the Bradford method. LR – lipid raft. Non-LR – non raft fraction. WT – wild type.
